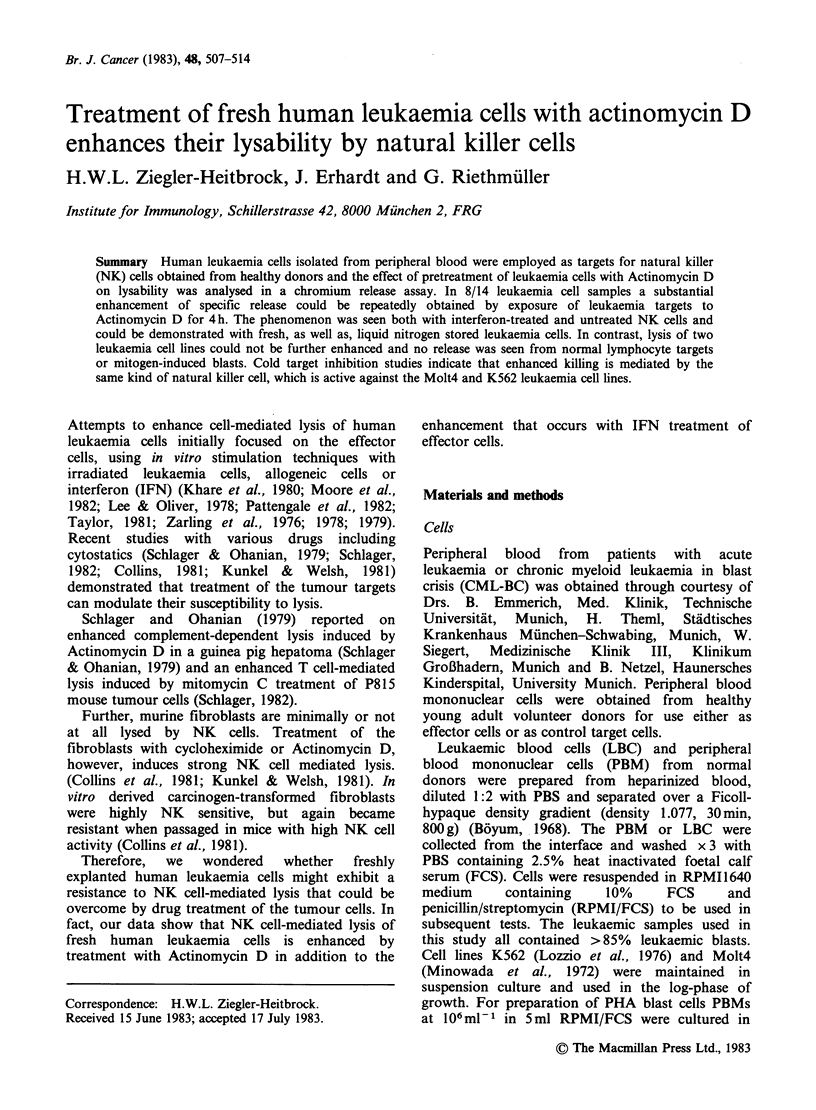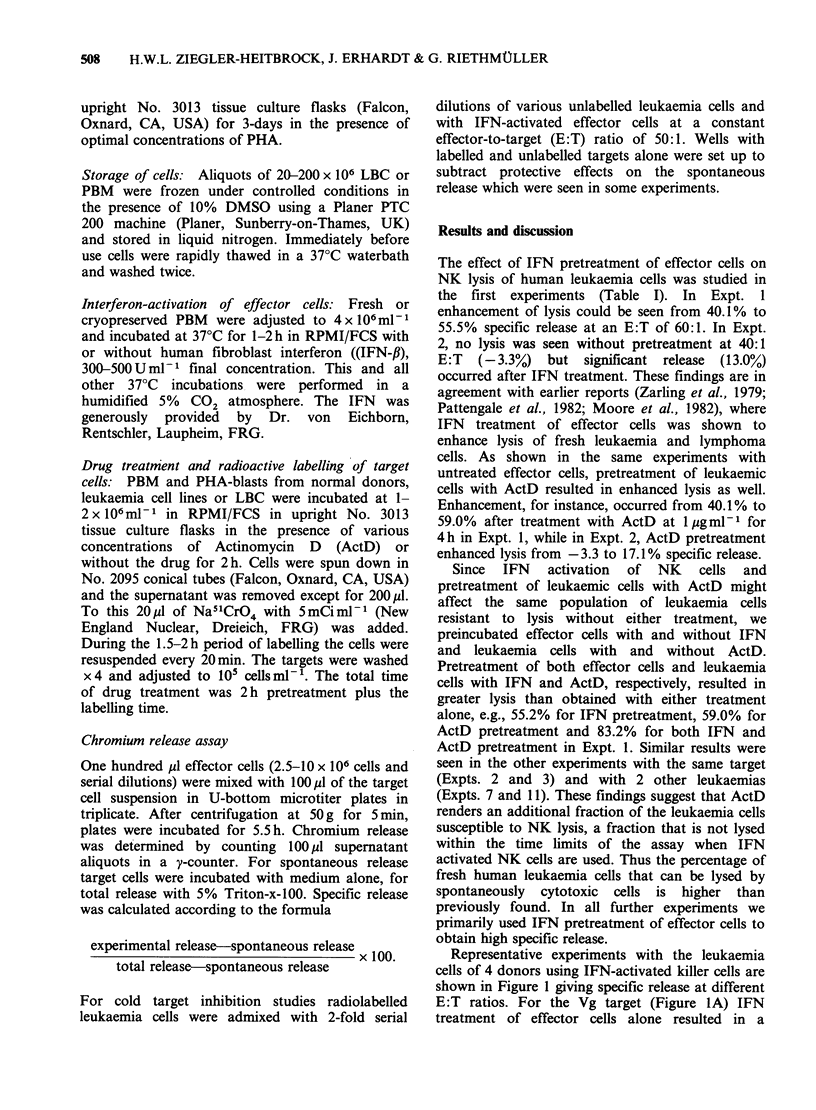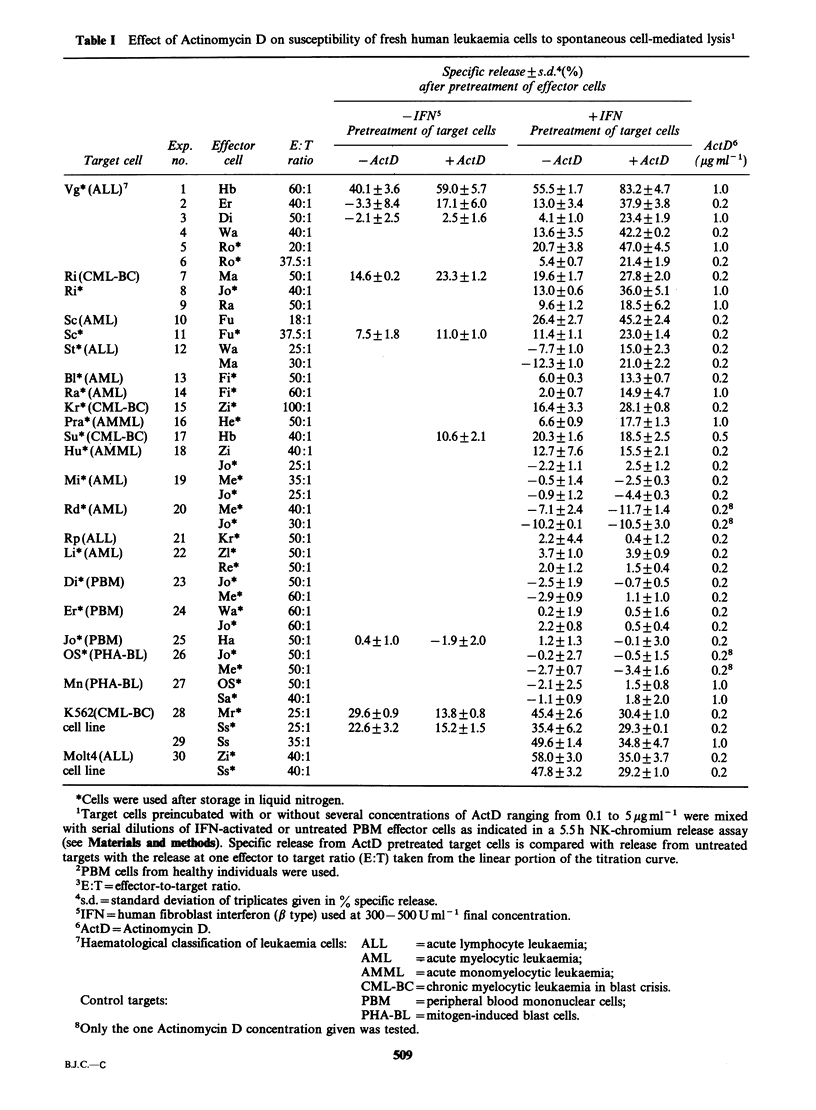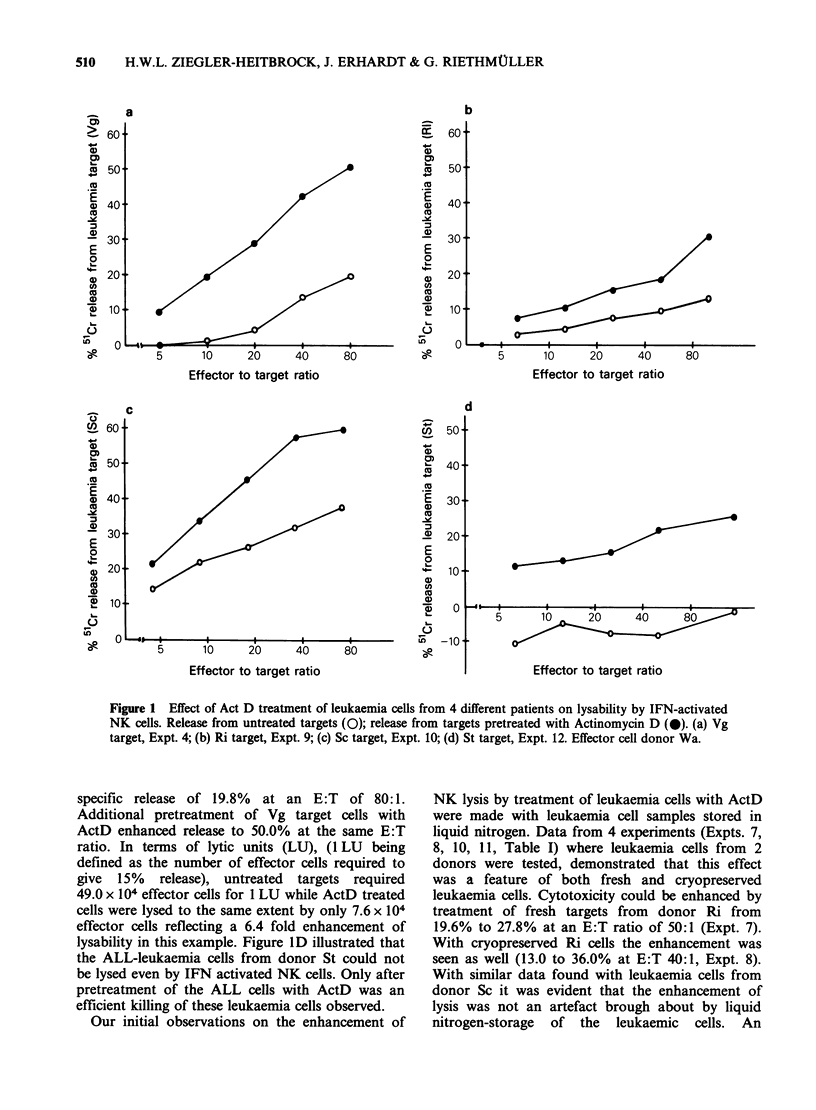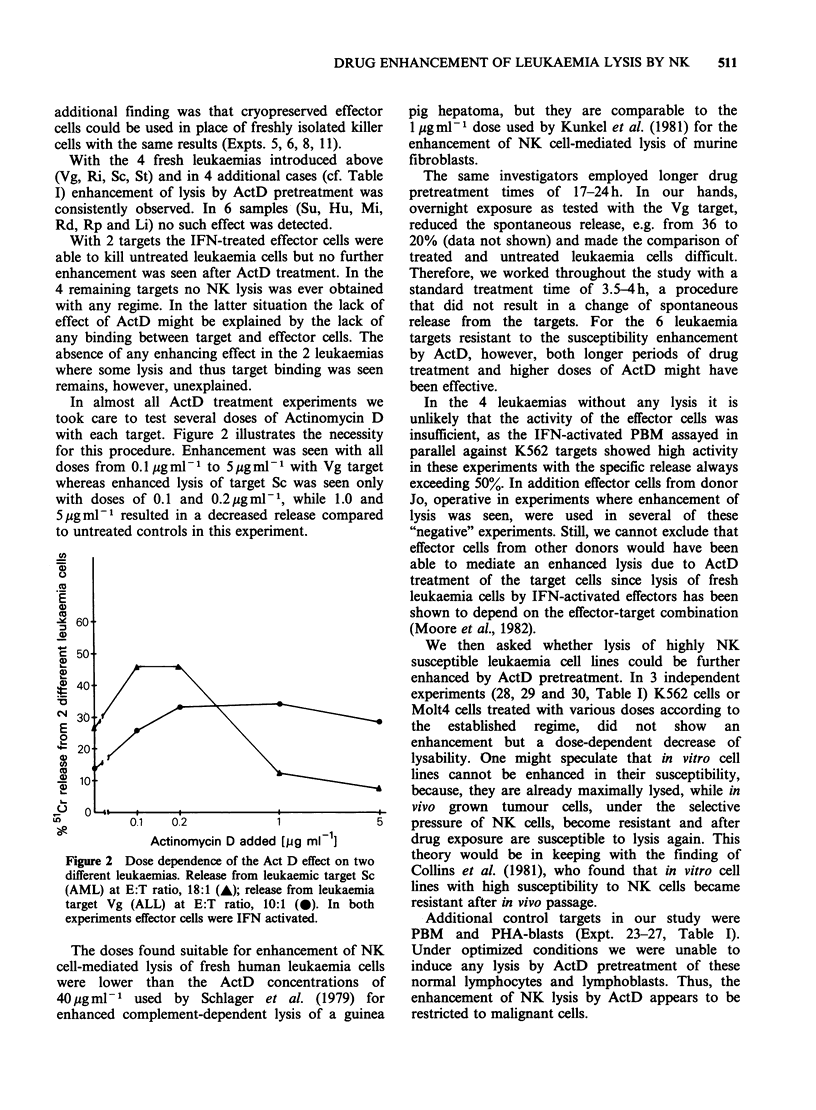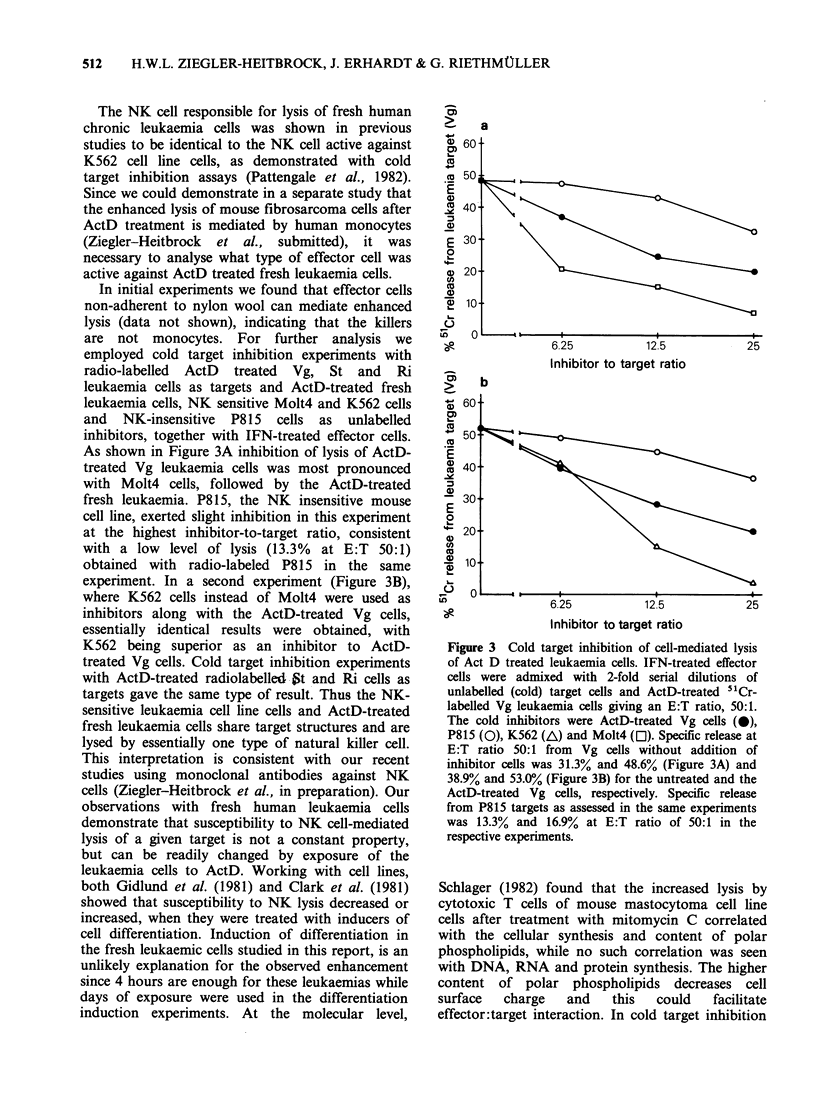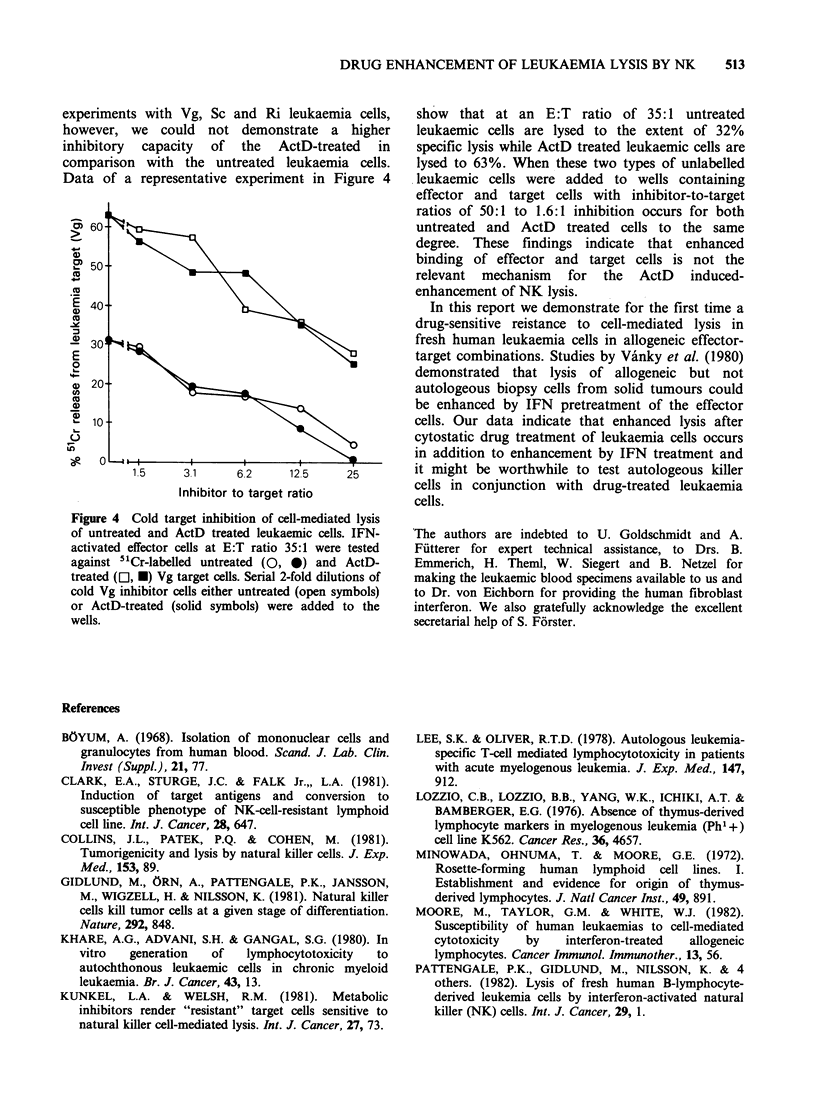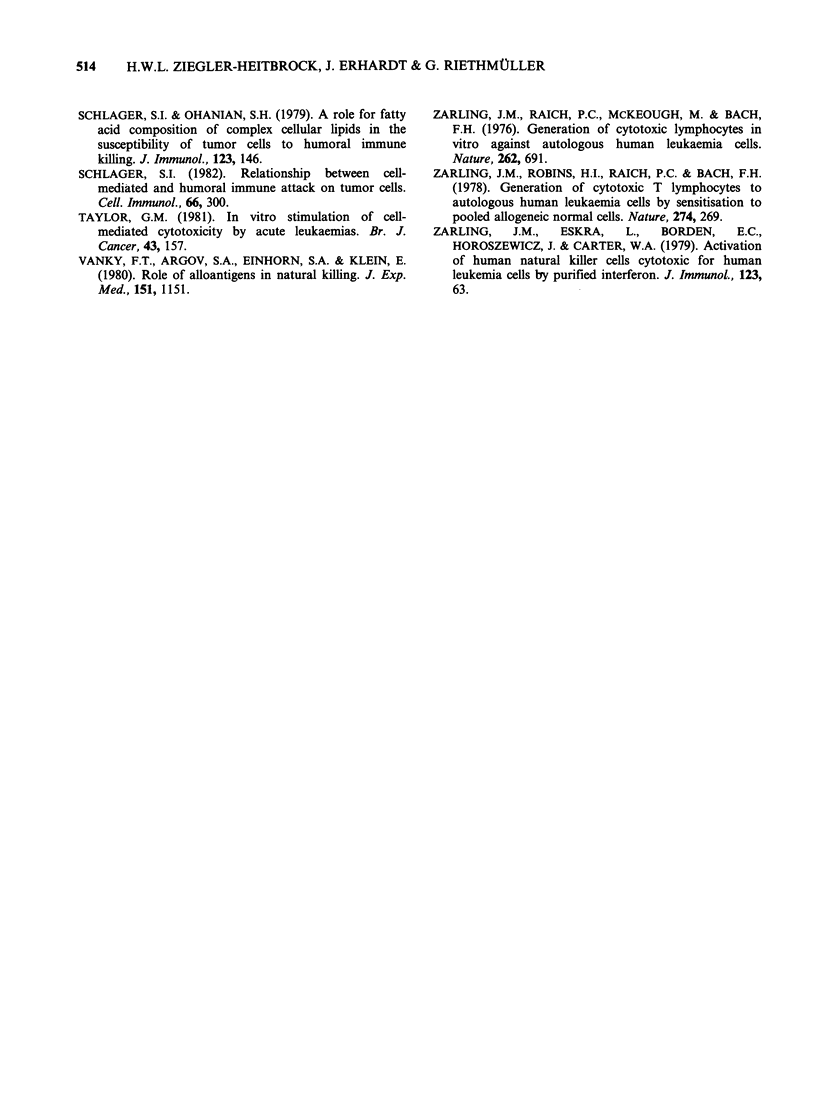# Treatment of fresh human leukaemia cells with actinomycin D enhances their lysability by natural killer cells.

**DOI:** 10.1038/bjc.1983.223

**Published:** 1983-10

**Authors:** H. W. Ziegler-Heitbrock, J. Erhardt, G. Riethmüller

## Abstract

Human leukaemia cells isolated from peripheral blood were employed as targets for natural killer (NK) cells obtained from healthy donors and the effect of pretreatment of leukaemia cells with Actinomycin D on lysability was analysed in a chromium release assay. In 8/14 leukaemia cell samples a substantial enhancement of specific release could be repeatedly obtained by exposure of leukaemia targets to Actinomycin D for 4 h. The phenomenon was seen both with interferon-treated and untreated NK cells and could be demonstrated with fresh, as well as, liquid nitrogen stored leukaemia cells. In contrast, lysis of two leukaemia cell lines could not be further enhanced and no release was seen from normal lymphocyte targets or mitogen-induced blasts. Cold target inhibition studies indicate that enhanced killing is mediated by the same kind of natural killer cell, which is active against the Molt4 and K562 leukaemia cell lines.


*Br. J. Cancer* (1983), **48**, 507–514

### Treatment of fresh human leukaemia cells with actinomycin D enhances their lysability by natural killer cells

H.W.L. Ziegler-Heitbrock, J. Erhardt and G. Riethmüller

*Institute for Immunology, Schillerstrasse 42, 8000 München 2, FRG*

**Summary** Human leukaemia cells isolated from peripheral blood were employed as targets for natural killer (NK) cells obtained from healthy donors and the effect of pretreatment of leukaemia cells with Actinomycin D on lysability was analysed in a chromium release assay. In 8/14 leukaemia cell samples a substantial enhancement of specific release could be repeatedly obtained by exposure of leukaemia targets to Actinomycin D for 4 h. The phenomenon was seen both with interferon-treated and untreated NK cells and could be demonstrated with fresh, as well as, liquid nitrogen stored leukaemia cells. In contrast, lysis of two leukaemia cell lines could not be further enhanced and no release was seen from normal lymphocyte targets or mitogen-induced blasts. Cold target inhibition studies indicate that enhanced killing is mediated by the same kind of natural killer cell, which is active against the Molt4 and K562 leukaemia cell lines.

Attempts to enhance cell-mediated lysis of human leukaemia cells initially focused on the effector cells, using *in vitro* stimulation techniques with irradiated leukaemia cells, allogeneic cells or interferon (IFN) (Khare *et al.*, 1980; Moore *et al.*, 1982; Lee & Oliver, 1978; Pattengale *et al.*, 1982; Taylor, 1981; Zarling *et al.*, 1976; 1978; 1979). Recent studies with various drugs including cytostatics (Schlager & Ohanian, 1979; Schlager, 1982; Collins, 1981; Kunkel & Welsh, 1981) demonstrated that treatment of the tumour targets can modulate their susceptibility to lysis.

Schlager and Ohanian (1979) reported on enhanced complement-dependent lysis induced by Actinomycin D in a guinea pig hepatoma (Schlager & Ohanian, 1979) and an enhanced T cell-mediated lysis induced by mitomycin C treatment of P815 mouse tumour cells (Schlager, 1982).

Further, murine fibroblasts are minimally or not at all lysed by NK cells. Treatment of the fibroblasts with cycloheximide or Actinomycin D, however, induces strong NK cell mediated lysis. (Collins *et al.*, 1981; Kunkel & Welsh, 1981). *In vitro* derived carcinogen-transformed fibroblasts were highly NK sensitive, but again became resistant when passaged in mice with high NK cell activity (Collins *et al.*, 1981).

Therefore, we wondered whether freshly explanted human leukaemia cells might exhibit a resistance to NK cell-mediated lysis that could be overcome by drug treatment of the tumour cells. In fact, our data show that NK cell-mediated lysis of fresh human leukaemia cells is enhanced by treatment with Actinomycin D in addition to the enhancement that occurs with IFN treatment of effector cells.

Correspondence: H.W.L. Ziegler-Heitbrock.
Received 15 June 1983; accepted 17 July 1983.

### Materials and methods

### *Cells*

Peripheral blood from patients with acute leukaemia or chronic myeloid leukaemia in blast crisis (CML-BC) was obtained through courtesy of Drs. B. Emmerich, Med. Klinik, Technische Universität, Munich, H. Theml, Städtisches Krankenhaus München–Schwabing, Munich, W. Siegert, Medizinische Klinik III, Klinikum Großhadern, Munich and B. Netzel, Haunersches Kinderspital, University Munich. Peripheral blood mononuclear cells were obtained from healthy young adult volunteer donors for use either as effector cells or as control target cells.

Leukaemic blood cells (LBC) and peripheral blood mononuclear cells (PBM) from normal donors were prepared from heparinized blood, diluted 1:2 with PBS and separated over a Ficoll-hypaque density gradient (density 1.077, 30 min, 800 g) (Böyum, 1968). The PBM or LBC were collected from the interface and washed $\times 3$ with PBS containing 2.5% heat inactivated foetal calf serum (FCS). Cells were resuspended in RPMI1640 medium containing 10% FCS and penicillin/streptomycin (RPMI/FCS) to be used in subsequent tests. The leukaemic samples used in this study all contained >85% leukaemic blasts. Cell lines K562 (Lozzio *et al.*, 1976) and Molt4 (Minowada *et al.*, 1972) were maintained in suspension culture and used in the log-phase of growth. For preparation of PHA blast cells PBMs at $10^6 ml^{-1}$ in 5 ml RPMI/FCS were cultured in

© The Macmillan Press Ltd., 1983

upright No. 3013 tissue culture flasks (Falcon, Oxnard, CA, USA) for 3-days in the presence of optimal concentrations of PHA.

*Storage of cells:* Aliquots of $20–200 \times 10^6$ LBC or PBM were frozen under controlled conditions in the presence of 10% DMSO using a Planer PTC 200 machine (Planer, Sunberry-on-Thames, UK) and stored in liquid nitrogen. Immediately before use cells were rapidly thawed in a 37°C waterbath and washed twice.

*Interferon-activation of effector cells:* Fresh or cryopreserved PBM were adjusted to $4 \times 10^6 ml^{-1}$ and incubated at 37°C for 1–2 h in RPMI/FCS with or without human fibroblast interferon ((IFN-$\beta$), 300–500 U $ml^{-1}$ final concentration. This and all other 37°C incubations were performed in a humidified 5% $CO_2$ atmosphere. The IFN was generously provided by Dr. von Eichborn, Rentschler, Laupheim, FRG.

*Drug treatment and radioactive labelling of target cells:* PBM and PHA-blasts from normal donors, leukaemia cell lines or LBC were incubated at $1–2 \times 10^6 ml^{-1}$ in RPMI/FCS in upright No. 3013 tissue culture flasks in the presence of various concentrations of Actinomycin D (ActD) or without the drug for 2h. Cells were spun down in No. 2095 conical tubes (Falcon, Oxnard, CA, USA) and the supernatant was removed except for 200 $\mu$l. To this 20 $\mu$l of $Na^{51}CrO_4$ with 5 mCi $ml^{-1}$ (New England Nuclear, Dreieich, FRG) was added. During the 1.5–2 h period of labelling the cells were resuspended every 20 min. The targets were washed $\times 4$ and adjusted to $10^5$ cells $ml^{-1}$. The total time of drug treatment was 2 h pretreatment plus the labelling time.

### *Chromium release assay*

One hundred $\mu$l effector cells ($2.5–10 \times 10^6$ cells and serial dilutions) were mixed with 100 $\mu$l of the target cell suspension in U-bottom microtiter plates in triplicate. After centrifugation at 50 g for 5 min, plates were incubated for 5.5 h. Chromium release was determined by counting 100 $\mu$l supernatant aliquots in a $\gamma$-counter. For spontaneous release target cells were incubated with medium alone, for total release with 5% Triton-x-100. Specific release was calculated according to the formula

$$\frac{\text{experimental release—spontaneous release}}{\text{total release—spontaneous release}} \times 100.$$

For cold target inhibition studies radiolabelled leukaemia cells were admixed with 2-fold serial dilutions of various unlabelled leukaemia cells and with IFN-activated effector cells at a constant effector-to-target (E:T) ratio of 50:1. Wells with labelled and unlabelled targets alone were set up to subtract protective effects on the spontaneous release which were seen in some experiments.

### Results and discussion

The effect of IFN pretreatment of effector cells on NK lysis of human leukaemia cells was studied in the first experiments (Table I). In Expt. 1 enhancement of lysis could be seen from 40.1% to 55.5% specific release at an E:T of 60:1. In Expt. 2, no lysis was seen without pretreatment at 40:1 E:T (−3.3%) but significant release (13.0%) occurred after IFN treatment. These findings are in agreement with earlier reports (Zarling *et al.*, 1979; Pattengale *et al.*, 1982; Moore *et al.*, 1982), where IFN treatment of effector cells was shown to enhance lysis of fresh leukaemia and lymphoma cells. As shown in the same experiments with untreated effector cells, pretreatment of leukaemic cells with ActD resulted in enhanced lysis as well. Enhancement, for instance, occurred from 40.1% to 59.0% after treatment with ActD at 1 $\mu g ml^{-1}$ for 4 h in Expt. 1, while in Expt. 2, ActD pretreatment enhanced lysis from −3.3 to 17.1% specific release.

Since IFN activation of NK cells and pretreatment of leukaemic cells with ActD might affect the same population of leukaemia cells resistant to lysis without either treatment, we preincubated effector cells with and without IFN and leukaemia cells with and without ActD. Pretreatment of both effector cells and leukaemia cells with IFN and ActD, respectively, resulted in greater lysis than obtained with either treatment alone, e.g., 55.2% for IFN pretreatment, 59.0% for ActD pretreatment and 83.2% for both IFN and ActD pretreatment in Expt. 1. Similar results were seen in the other experiments with the same target (Expts. 2 and 3) and with 2 other leukaemias (Expts. 7 and 11). These findings suggest that ActD renders an additional fraction of the leukaemia cells susceptible to NK lysis, a fraction that is not lysed within the time limits of the assay when IFN activated NK cells are used. Thus the percentage of fresh human leukaemia cells that can be lysed by spontaneously cytotoxic cells is higher than previously found. In all further experiments we primarily used IFN pretreatment of effector cells to obtain high specific release.

Representative experiments with the leukaemia cells of 4 donors using IFN-activated killer cells are shown in Figure 1 giving specific release at different E:T ratios. For the Vg target (Figure 1A) IFN treatment of effector cells alone resulted in a

**Table I** Effect of Actinomycin D on susceptibility of fresh human leukaemia cells to spontaneous cell-mediated lysis[1]

| | | | | *Specific release* $\pm$ *s.d.*[4] *(%) after pretreatment of effector cells* | | | | |
|---|---|---|---|---|---|---|---|---|
| | | | | *−IFN*[5] *Pretreatment of target cells* | | *+IFN Pretreatment of target cells* | | |
| *Target cell* | *Exp. no.* | *Effector cell* | *E:T ratio* | *−ActD* | *+ActD* | *−ActD* | *+ActD* | *ActD*[6] *(µg ml$^{-1}$)* |
| Vg* (ALL)[7] | 1 | Hb | 60:1 | 40.1 ± 3.6 | 59.0 ± 5.7 | 55.5 ± 1.7 | 83.2 ± 4.7 | 1.0 |
| | 2 | Er | 40:1 | −3.3 ± 8.4 | 17.1 ± 6.0 | 13.0 ± 3.4 | 37.9 ± 3.8 | 0.2 |
| | 3 | Di | 50:1 | −2.1 ± 2.5 | 2.5 ± 1.6 | 4.1 ± 1.0 | 23.4 ± 1.9 | 1.0 |
| | 4 | Wa | 40:1 | | | 13.6 ± 3.5 | 42.2 ± 0.2 | 0.2 |
| | 5 | Ro* | 20:1 | | | 20.7 ± 3.8 | 47.0 ± 4.5 | 1.0 |
| | 6 | Ro* | 37.5:1 | | | 5.4 ± 0.7 | 21.4 ± 1.9 | 0.2 |
| Ri (CML-BC) | 7 | Ma | 50:1 | 14.6 ± 0.2 | 23.3 ± 1.2 | 19.6 ± 1.7 | 27.8 ± 2.0 | 0.2 |
| Ri* | 8 | Jo* | 40:1 | | | 13.0 ± 0.6 | 36.0 ± 5.1 | 1.0 |
| | 9 | Ra | 50:1 | | | 9.6 ± 1.2 | 18.5 ± 6.2 | 1.0 |
| Sc (AML) | 10 | Fu | 18:1 | | | 26.4 ± 2.7 | 45.2 ± 2.4 | 0.2 |
| Sc* | 11 | Fu* | 37.5:1 | 7.5 ± 1.8 | 11.0 ± 1.0 | 11.4 ± 1.1 | 23.0 ± 1.4 | 0.2 |
| St* (ALL) | 12 | Wa | 25:1 | | | −7.7 ± 1.0 | 15.0 ± 2.3 | 0.2 |
| | | Ma | 30:1 | | | −12.3 ± 1.0 | 21.0 ± 2.2 | 0.2 |
| Bl* (AML) | 13 | Fi* | 50:1 | | | 6.0 ± 0.3 | 13.3 ± 0.7 | 0.2 |
| Ra* (AML) | 14 | Fi* | 60:1 | | | 2.0 ± 0.7 | 14.9 ± 4.7 | 1.0 |
| Kr* (CML-BC) | 15 | Zi* | 100:1 | | | 16.4 ± 3.3 | 28.1 ± 0.8 | 0.2 |
| Pra* (AMML) | 16 | He* | 50:1 | | | 6.6 ± 0.9 | 17.7 ± 1.3 | 1.0 |
| Su* (CML-BC) | 17 | Hb | 40:1 | | 10.6 ± 2.1 | 20.3 ± 1.6 | 18.5 ± 2.5 | 0.5 |
| Hu* (AMML) | 18 | Zi | 40:1 | | | 12.7 ± 7.6 | 15.5 ± 2.1 | 0.2 |
| | | Jo* | 25:1 | | | −2.2 ± 1.1 | 2.5 ± 1.2 | 0.2 |
| Mi* (AML) | 19 | Me* | 35:1 | | | −0.5 ± 1.4 | −2.5 ± 0.3 | 0.2 |
| | | Jo* | 25:1 | | | −0.9 ± 1.2 | −4.4 ± 0.3 | 0.2 |
| Rd* (AML) | 20 | Me* | 40:1 | | | −7.1 ± 2.4 | −11.7 ± 1.4 | 0.2[8] |
| | | Jo* | 30:1 | | | −10.2 ± 0.1 | −10.5 ± 3.0 | 0.2[8] |
| Rp (ALL) | 21 | Kr* | 50:1 | | | 2.2 ± 4.4 | 0.4 ± 1.2 | 0.2 |
| Li* (AML) | 22 | Zl* | 50:1 | | | 3.7 ± 1.0 | 3.9 ± 0.9 | 0.2 |
| | | Re* | 50:1 | | | 2.0 ± 1.2 | 1.5 ± 0.4 | 0.2 |
| Di* (PBM) | 23 | Jo* | 50:1 | | | −2.5 ± 1.9 | −0.7 ± 0.5 | 0.2 |
| | | Me* | 60:1 | | | −2.9 ± 0.9 | 1.1 ± 1.0 | 0.2 |
| Er* (PBM) | 24 | Wa* | 60:1 | | | 0.2 ± 1.9 | 0.5 ± 1.6 | 0.2 |
| | | Jo* | 60:1 | | | 2.2 ± 0.8 | 0.5 ± 0.4 | 0.2 |
| Jo* (PBM) | 25 | Ha | 50:1 | 0.4 ± 1.0 | −1.9 ± 2.0 | 1.2 ± 1.3 | −0.1 ± 3.0 | 0.2 |
| OS* (PHA-BL) | 26 | Jo* | 50:1 | | | −0.2 ± 2.7 | −0.5 ± 1.5 | 0.2[8] |
| | | Me* | 50:1 | | | −2.7 ± 0.7 | −3.4 ± 1.6 | 0.2[8] |
| Mn (PHA-BL) | 27 | OS* | 50:1 | | | −2.1 ± 2.5 | 1.5 ± 0.8 | 1.0 |
| | | Sa* | 40:1 | | | −1.1 ± 0.9 | 1.8 ± 2.0 | 1.0 |
| K562 (CML-BC) | 28 | Mr* | 25:1 | 29.6 ± 0.9 | 13.8 ± 0.8 | 45.4 ± 2.6 | 30.4 ± 1.0 | 0.2 |
| cell line | | Ss* | 25:1 | 22.6 ± 3.2 | 15.2 ± 1.5 | 35.4 ± 6.2 | 29.3 ± 0.1 | 0.2 |
| | 29 | Ss | 35:1 | | | 49.6 ± 1.4 | 34.8 ± 4.7 | 1.0 |
| Molt4 (ALL) | 30 | Zi* | 40:1 | | | 58.0 ± 3.0 | 35.0 ± 3.7 | 0.2 |
| cell line | | Ss* | 40:1 | | | 47.8 ± 3.2 | 29.2 ± 1.0 | 0.2 |

*Cells were used after storage in liquid nitrogen.

[1]Target cells preincubated with or without several concentrations of ActD ranging from 0.1 to 5 µg ml$^{-1}$ were mixed with serial dilutions of IFN-activated or untreated PBM effector cells as indicated in a 5.5 h NK-chromium release assay (see **Materials and methods**). Specific release from ActD pretreated target cells is compared with release from untreated targets with the release at one effector to target ratio (E:T) taken from the linear portion of the titration curve.

[2]PBM cells from healthy individuals were used.

[3]E:T = effector-to-target ratio.

[4]s.d. = standard deviation of triplicates given in % specific release.

[5]IFN = human fibroblast interferon (β type) used at 300 − 500 U ml$^{-1}$ final concentration.

[6]ActD = Actinomycin D.

[7]Haematological classification of leukaemia cells:
ALL = acute lymphocyte leukaemia;
AML = acute myelocytic leukaemia;
AMML = acute monomyelocytic leukaemia;
CML-BC = chronic myelocytic leukaemia in blast crisis.
Control targets:
PBM = peripheral blood mononuclear cells;
PHA-BL = mitogen-induced blast cells.

[8]Only the one Actinomycin D concentration given was tested.

**Figure 1** Effect of Act D treatment of leukaemia cells from 4 different patients on lysability by IFN-activated NK cells. Release from untreated targets (O); release from targets pretreated with Actinomycin D (●). (a) Vg target, Expt. 4; (b) Ri target, Expt. 9; (c) Sc target, Expt. 10; (d) St target, Expt. 12. Effector cell donor Wa.

specific release of 19.8% at an E:T of 80:1. Additional pretreatment of Vg target cells with ActD enhanced release to 50.0% at the same E:T ratio. In terms of lytic units (LU), (1 LU being defined as the number of effector cells required to give 15% release), untreated targets required $49.0 \times 10^4$ effector cells for 1 LU while ActD treated cells were lysed to the same extent by only $7.6 \times 10^4$ effector cells reflecting a 6.4 fold enhancement of lysability in this example. Figure 1D illustrated that the ALL-leukaemia cells from donor St could not be lysed even by IFN activated NK cells. Only after pretreatment of the ALL cells with ActD was an efficient killing of these leukaemia cells observed.

Our initial observations on the enhancement of NK lysis by treatment of leukaemia cells with ActD were made with leukaemia cell samples stored in liquid nitrogen. Data from 4 experiments (Expts. 7, 8, 10, 11, Table I) where leukaemia cells from 2 donors were tested, demonstrated that this effect was a feature of both fresh and cryopreserved leukaemia cells. Cytotoxicity could be enhanced by treatment of fresh targets from donor Ri from 19.6% to 27.8% at an E:T ratio of 50:1 (Expt. 7). With cryopreserved Ri cells the enhancement was seen as well (13.0 to 36.0% at E:T 40:1, Expt. 8). With similar data found with leukaemia cells from donor Sc it was evident that the enhancement of lysis was not an artefact brough about by liquid nitrogen-storage of the leukaemic cells. An additional finding was that cryopreserved effector cells could be used in place of freshly isolated killer cells with the same results (Expts. 5, 6, 8, 11).

With the 4 fresh leukaemias introduced above (Vg, Ri, Sc, St) and in 4 additional cases (cf. Table I) enhancement of lysis by ActD pretreatment was consistently observed. In 6 samples (Su, Hu, Mi, Rd, Rp and Li) no such effect was detected.

With 2 targets the IFN-treated effector cells were able to kill untreated leukaemia cells but no further enhancement was seen after ActD treatment. In the 4 remaining targets no NK lysis was ever obtained with any regime. In the latter situation the lack of effect of ActD might be explained by the lack of any binding between target and effector cells. The absence of any enhancing effect in the 2 leukaemias where some lysis and thus target binding was seen remains, however, unexplained.

In almost all ActD treatment experiments we took care to test several doses of Actinomycin D with each target. Figure 2 illustrates the necessity for this procedure. Enhancement was seen with all doses from 0.1 $\mu g ml^{-1}$ to 5 $\mu g ml^{-1}$ with Vg target whereas enhanced lysis of target Sc was seen only with doses of 0.1 and 0.2 $\mu g ml^{-1}$, while 1.0 and 5 $\mu g ml^{-1}$ resulted in a decreased release compared to untreated controls in this experiment.

**Figure 2** Dose dependence of the Act D effect on two different leukaemias. Release from leukaemic target Sc (AML) at E:T ratio, 18:1 (▲); release from leukaemia target Vg (ALL) at E:T ratio, 10:1 (●). In both experiments effector cells were IFN activated.

The doses found suitable for enhancement of NK cell-mediated lysis of fresh human leukaemia cells were lower than the ActD concentrations of 40 $\mu g ml^{-1}$ used by Schlager *et al.* (1979) for enhanced complement-dependent lysis of a guinea pig hepatoma, but they are comparable to the 1 $\mu g ml^{-1}$ dose used by Kunkel *et al.* (1981) for the enhancement of NK cell-mediated lysis of murine fibroblasts.

The same investigators employed longer drug pretreatment times of 17–24 h. In our hands, overnight exposure as tested with the Vg target, reduced the spontaneous release, e.g. from 36 to 20% (data not shown) and made the comparison of treated and untreated leukaemia cells difficult. Therefore, we worked throughout the study with a standard treatment time of 3.5–4 h, a procedure that did not result in a change of spontaneous release from the targets. For the 6 leukaemia targets resistant to the susceptibility enhancement by ActD, however, both longer periods of drug treatment and higher doses of ActD might have been effective.

In the 4 leukaemias without any lysis it is unlikely that the activity of the effector cells was insufficient, as the IFN-activated PBM assayed in parallel against K562 targets showed high activity in these experiments with the specific release always exceeding 50%. In addition effector cells from donor Jo, operative in experiments where enhancement of lysis was seen, were used in several of these "negative" experiments. Still, we cannot exclude that effector cells from other donors would have been able to mediate an enhanced lysis due to ActD treatment of the target cells since lysis of fresh leukaemia cells by IFN-activated effectors has been shown to depend on the effector-target combination (Moore *et al.*, 1982).

We then asked whether lysis of highly NK susceptible leukaemia cell lines could be further enhanced by ActD pretreatment. In 3 independent experiments (28, 29 and 30, Table I) K562 cells or Molt4 cells treated with various doses according to the established regime, did not show an enhancement but a dose-dependent decrease of lysability. One might speculate that *in vitro* cell lines cannot be enhanced in their susceptibility, because, they are already maximally lysed, while *in vivo* grown tumour cells, under the selective pressure of NK cells, become resistant and after drug exposure are susceptible to lysis again. This theory would be in keeping with the finding of Collins *et al.* (1981), who found that *in vitro* cell lines with high susceptibility to NK cells became resistant after *in vivo* passage.

Additional control targets in our study were PBM and PHA-blasts (Expt. 23–27, Table I). Under optimized conditions we were unable to induce any lysis by ActD pretreatment of these normal lymphocytes and lymphoblasts. Thus, the enhancement of NK lysis by ActD appears to be restricted to malignant cells.

The NK cell responsible for lysis of fresh human chronic leukaemia cells was shown in previous studies to be identical to the NK cell active against K562 cell line cells, as demonstrated with cold target inhibition assays (Pattengale *et al.*, 1982). Since we could demonstrate in a separate study that the enhanced lysis of mouse fibrosarcoma cells after ActD treatment is mediated by human monocytes (Ziegler–Heitbrock *et al.*, submitted), it was necessary to analyse what type of effector cell was active against ActD treated fresh leukaemia cells.

In initial experiments we found that effector cells non-adherent to nylon wool can mediate enhanced lysis (data not shown), indicating that the killers are not monocytes. For further analysis we employed cold target inhibition experiments with radio-labelled ActD treated Vg, St and Ri leukaemia cells as targets and ActD-treated fresh leukaemia cells, NK sensitive Molt4 and K562 cells and NK-insensitive P815 cells as unlabelled inhibitors, together with IFN-treated effector cells. As shown in Figure 3A inhibition of lysis of ActD-treated Vg leukaemia cells was most pronounced with Molt4 cells, followed by the ActD-treated fresh leukaemia. P815, the NK insensitive mouse cell line, exerted slight inhibition in this experiment at the highest inhibitor-to-target ratio, consistent with a low level of lysis (13.3% at E:T 50:1) obtained with radio-labeled P815 in the same experiment. In a second experiment (Figure 3B), where K562 cells instead of Molt4 were used as inhibitors along with the ActD-treated Vg cells, essentially identical results were obtained, with K562 being superior as an inhibitor to ActD-treated Vg cells. Cold target inhibition experiments with ActD-treated radiolabelled St and Ri cells as targets gave the same type of result. Thus the NK-sensitive leukaemia cell line cells and ActD-treated fresh leukaemia cells share target structures and are lysed by essentially one type of natural killer cell. This interpretation is consistent with our recent studies using monoclonal antibodies against NK cells (Ziegler–Heitbrock *et al.*, in preparation). Our observations with fresh human leukaemia cells demonstrate that susceptibility to NK cell-mediated lysis of a given target is not a constant property, but can be readily changed by exposure of the leukaemia cells to ActD. Working with cell lines, both Gidlund *et al.* (1981) and Clark *et al.* (1981) showed that susceptibility to NK lysis decreased or increased, when they were treated with inducers of cell differentiation. Induction of differentiation in the fresh leukaemic cells studied in this report, is an unlikely explanation for the observed enhancement since 4 hours are enough for these leukaemias while days of exposure were used in the differentiation induction experiments. At the molecular level,

**Figure 3** Cold target inhibition of cell-mediated lysis of Act D treated leukaemia cells. IFN-treated effector cells were admixed with 2-fold serial dilutions of unlabelled (cold) target cells and ActD-treated $^{51}$Cr-labelled Vg leukaemia cells giving an E:T ratio, 50:1. The cold inhibitors were ActD-treated Vg cells (●), P815 (○), K562 (△) and Molt4 (□). Specific release at E:T ratio 50:1 from Vg cells without addition of inhibitor cells was 31.3% and 48.6% (Figure 3A) and 38.9% and 53.0% (Figure 3B) for the untreated and the ActD-treated Vg cells, respectively. Specific release from P815 targets as assessed in the same experiments was 13.3% and 16.9% at E:T ratio of 50:1 in the respective experiments.

Schlager (1982) found that the increased lysis by cytotoxic T cells of mouse mastocytoma cell line cells after treatment with mitomycin C correlated with the cellular synthesis and content of polar phospholipids, while no such correlation was seen with DNA, RNA and protein synthesis. The higher content of polar phospholipids decreases cell surface charge and this could facilitate effector:target interaction. In cold target inhibition experiments with Vg, Sc and Ri leukaemia cells, however, we could not demonstrate a higher inhibitory capacity of the ActD-treated in comparison with the untreated leukaemia cells. Data of a representative experiment in Figure 4

**Figure 4** Cold target inhibition of cell-mediated lysis of untreated and ActD treated leukaemic cells. IFN-activated effector cells at E:T ratio 35:1 were tested against $^{51}$Cr-labelled untreated (○, ●) and ActD-treated (□, ■) Vg target cells. Serial 2-fold dilutions of cold Vg inhibitor cells either untreated (open symbols) or ActD-treated (solid symbols) were added to the wells.

show that at an E:T ratio of 35:1 untreated leukaemic cells are lysed to the extent of 32% specific lysis while ActD treated leukaemic cells are lysed to 63%. When these two types of unlabelled leukaemic cells were added to wells containing effector and target cells with inhibitor-to-target ratios of 50:1 to 1.6:1 inhibition occurs for both untreated and ActD treated cells to the same degree. These findings indicate that enhanced binding of effector and target cells is not the relevant mechanism for the ActD induced-enhancement of NK lysis.

In this report we demonstrate for the first time a drug-sensitive reistance to cell-mediated lysis in fresh human leukaemia cells in allogeneic effector-target combinations. Studies by Vânky *et al.* (1980) demonstrated that lysis of allogeneic but not autologeous biopsy cells from solid tumours could be enhanced by IFN pretreatment of the effector cells. Our data indicate that enhanced lysis after cytostatic drug treatment of leukaemia cells occurs in addition to enhancement by IFN treatment and it might be worthwhile to test autologeous killer cells in conjunction with drug-treated leukaemia cells.

The authors are indebted to U. Goldschmidt and A. Fütterer for expert technical assistance, to Drs. B. Emmerich, H. Theml, W. Siegert and B. Netzel for making the leukaemic blood specimens available to us and to Dr. von Eichborn for providing the human fibroblast interferon. We also gratefully acknowledge the excellent secretarial help of S. Förster.

### References

BÖYUM, A. (1968). Isolation of mononuclear cells and granulocytes from human blood. *Scand. J. Lab. Clin. Invest (Suppl.)*, **21**, 77.

CLARK, E.A., STURGE, J.C. & FALK Jr.,, L.A. (1981). Induction of target antigens and conversion to susceptible phenotype of NK-cell-resistant lymphoid cell line. *Int. J. Cancer*, **28**, 647.

COLLINS, J.L., PATEK, P.Q. & COHEN, M. (1981). Tumorigenicity and lysis by natural killer cells. *J. Exp. Med.*, **153**, 89.

GIDLUND, M., ÖRN, A., PATTENGALE, P.K., JANSSON, M., WIGZELL, H. & NILSSON, K. (1981). Natural killer cells kill tumor cells at a given stage of differentiation. *Nature*, **292**, 848.

KHARE, A.G., ADVANI, S.H. & GANGAL, S.G. (1980). In vitro generation of lymphocytotoxicity to autochthonous leukaemic cells in chronic myeloid leukaemia. *Br. J. Cancer*, **43**, 13.

KUNKEL, L.A. & WELSH, R.M. (1981). Metabolic inhibitors render "resistant" target cells sensitive to natural killer cell-mediated lysis. *Int. J. Cancer*, **27**, 73.

LEE, S.K. & OLIVER, R.T.D. (1978). Autologous leukemia-specific T-cell mediated lymphocytotoxicity in patients with acute myelogenous leukemia. *J. Exp. Med.*, **147**, 912.

LOZZIO, C.B., LOZZIO, B.B., YANG, W.K., ICHIKI, A.T. & BAMBERGER, E.G. (1976). Absence of thymus-derived lymphocyte markers in myelogenous leukemia (Ph$^1$+) cell line K562. *Cancer Res.*, **36**, 4657.

MINOWADA, OHNUMA, T. & MOORE, G.E. (1972). Rosette-forming human lymphoid cell lines. I. Establishment and evidence for origin of thymus-derived lymphocytes. *J. Natl Cancer Inst.*, **49**, 891.

MOORE, M., TAYLOR, G.M. & WHITE, W.J. (1982). Susceptibility of human leukaemias to cell-mediated cytotoxicity by interferon-treated allogeneic lymphocytes. *Cancer Immunol. Immunother.*, **13**, 56.

PATTENGALE, P.K., GIDLUND, M., NILSSON, K. & 4 others. (1982). Lysis of fresh human B-lymphocyte-derived leukemia cells by interferon-activated natural killer (NK) cells. *Int. J. Cancer*, **29**, 1.

SCHLAGER, S.I. & OHANIAN, S.H. (1979). A role for fatty acid composition of complex cellular lipids in the susceptibility of tumor cells to humoral immune killing. *J. Immunol.*, **123**, 146.

SCHLAGER, S.I. (1982). Relationship between cell-mediated and humoral immune attack on tumor cells. *Cell. Immunol.*, **66**, 300.

TAYLOR, G.M. (1981). In vitro stimulation of cell-mediated cytotoxicity by acute leukaemias. *Br. J. Cancer*, **43**, 157.

VANKY, F.T., ARGOV, S.A., EINHORN, S.A. & KLEIN, E. (1980). Role of alloantigens in natural killing. *J. Exp. Med.*, **151**, 1151.

ZARLING, J.M., RAICH, P.C., MCKEOUGH, M. & BACH, F.H. (1976). Generation of cytotoxic lymphocytes in vitro against autologous human leukaemia cells. *Nature*, **262**, 691.

ZARLING, J.M., ROBINS, H.I., RAICH, P.C. & BACH, F.H. (1978). Generation of cytotoxic T lymphocytes to autologous human leukaemia cells by sensitisation to pooled allogeneic normal cells. *Nature*, **274**, 269.

ZARLING, J.M., ESKRA, L., BORDEN, E.C., HOROSZEWICZ, J. & CARTER, W.A. (1979). Activation of human natural killer cells cytotoxic for human leukemia cells by purified interferon. *J. Immunol.*, **123**, 63.